# Systems-Level Comparison of Host-Responses Elicited by Avian H5N1 and Seasonal H1N1 Influenza Viruses in Primary Human Macrophages

**DOI:** 10.1371/journal.pone.0008072

**Published:** 2009-12-14

**Authors:** Suki M. Y. Lee, Jennifer L. Gardy, C. Y. Cheung, Timothy K. W. Cheung, Kenrie P. Y. Hui, Nancy Y. Ip, Y. Guan, Robert E. W. Hancock, J. S. Malik Peiris

**Affiliations:** 1 Department of Microbiology, The University of Hong Kong, Hong Kong, China; 2 British Columbia Centre for Disease Control, Vancouver, British Columbia, Canada; 3 Department of Biochemistry, Biotechnology Research Institute, Hong Kong University of Science and Technology, Hong Kong, China; 4 Centre for Microbial Diseases and Immunity Research, University of British Columbia, Vancouver, British Columbia, Canada; 5 The University of Hong Kong-Pasteur Research Centre, Hong Kong, China; Charité-Universitätsmedizin Berlin, Germany

## Abstract

Human disease caused by highly pathogenic avian influenza (HPAI) H5N1 can lead to a rapidly progressive viral pneumonia leading to acute respiratory distress syndrome. There is increasing evidence from clinical, animal models and in vitro data, which suggests a role for virus-induced cytokine dysregulation in contributing to the pathogenesis of human H5N1 disease. The key target cells for the virus in the lung are the alveolar epithelium and alveolar macrophages, and we have shown that, compared to seasonal human influenza viruses, equivalent infecting doses of H5N1 viruses markedly up-regulate pro-inflammatory cytokines in both primary cell types in vitro. Whether this H5N1-induced dysregulation of host responses is driven by qualitative (i.e activation of unique host pathways in response to H5N1) or quantitative differences between seasonal influenza viruses is unclear. Here we used microarrays to analyze and compare the gene expression profiles in primary human macrophages at 1, 3, and 6 h after infection with H5N1 virus or low-pathogenic seasonal influenza A (H1N1) virus. We found that host responses to both viruses are qualitatively similar with the activation of nearly identical biological processes and pathways. However, in comparison to seasonal H1N1 virus, H5N1 infection elicits a quantitatively stronger host inflammatory response including type I interferon (IFN) and tumor necrosis factor (TNF)-α genes. A network-based analysis suggests that the synergy between IFN-β and TNF-α results in an enhanced and sustained IFN and pro-inflammatory cytokine response at the early stage of viral infection that may contribute to the viral pathogenesis and this is of relevance to the design of novel therapeutic strategies for H5N1 induced respiratory disease.

## Introduction

The emergence and spread of the highly pathogenic avian influenza virus (H5N1) in poultry and wild birds with repeated zoonotic transmission to humans has raised concerns about a possible pandemic [1]. Zoonotic H5N1 disease continues unabated in a number of countries and is likely grossly under-recognised. At the time of writing, 440 human cases have been reported with 262 fatalities, an overall case fatality rate of approximately 60% (Cumulative Number of Confirmed Human Cases of Avian Influenza A/H5N1 reported to World Health Organization). While a novel H1N1 virus is now spreading worldwide and has become pandemic, it remains relatively mild in its severity [2]. Given its origin from influenza viruses of swine [3], [4], there is a concern that this virus will become epizootic in pigs, similar to the 1918 pandemic H1N1 virus [5]. If so, there will be many opportunities for the pandemic H1N1 to reassort with avian H5N1, which has repeatedly been isolated from pigs [6]. Whether arising directly from the avian virus or through reassortment with a current human influenza virus (e.g. novel pandemic H1N1), an H5N1 pandemic remains a possibility. Although the risk of such an event is low, its potential impact is high, thus an understanding of the pathogenesis of human H5N1 disease remains a high priority.

A rapidly progressing primary viral pneumonia leading to acute respiratory distress syndrome is the primary cause of death in patients with H5N1 disease. The sustained high viral load in the lung, the tropism for the alveolar epithelium and the differential host responses to H5N1 viruses, individually or in combination, have been proposed as mechanism to explain the unusual virulence of this virus [7], [8]. Serum and lung of patients with H5N1 disease have markedly elevated levels of cytokines and chemokines [7], [9]. Cytokine dysreglation has also been seen in animals (mice, ferrets, macaques) experimentally infected with H5N1 virus when compared with seasonal influenza (H1N1 or H3N2) viruses [10], [11], [12]. We previously found that H5N1 viruses hyper-induce pro-inflammatory cytokines and chemokines in primary human macrophages and alveolar epithelium infected in vitro compared to a similar infecting dose of seasonal H1N1 virus [13], [14], [15], suggesting that differential host responses initiated by the H5N1 virus may contribute to the pathogenesis of H5N1 viruses in humans. In the in vitro models, using comparable infectious doses and by quantitating host responses at defined time points in a synchronous infection, it was possible to confirm that the difference in host responses between H5N1 and seasonal influenza are not merely reflections of the higher levels of virus replication, but reflect intrinsic differences of the virus. However, the mechanisms by which the H5N1 virus elicts these differential host respones are unclear.

Alveolar epithelial cells and macrophages are key target cells of the H5N1 virus in the lung [16]. Macrophages are also known to play a major amplifying role in H5N1 virus-induced cytokine cascades [15]. In this study, we have used primary human macrophages and analysed the early host response induced by H5N1 and H1N1 viruses at 1, 3 and 6 h post-infection by employing comprehensive gene expression profiling using an Affymetrix microarray platform. In particular, we aimed to provide insights into the mechanistic differences in host responses induced by these two viruses.

## Results

### H5N1 and H1N1 Influenza Virus-Elicited Host Responses in Human Monocyte-Derived Macrophages Are Qualitatively Similar but Quantitatively Different

We used the Affymetrix GeneChip Human Gene 1.0 ST array to compare the global gene expression profiles of human macrophages infected with H5N1, H1N1 viruses and mock-infected control cells at 1, 3 and 6 h post-infection. Changes were observed in 834 transcripts from 770 individual host genes (p<0.05 in 2 way ANOVA test).

In a preliminary analysis, gene expression data was analyzed from each macrophage donor separately to define the donor-to-donor variation after influenza infection. We used a ±2-fold change in gene expression as the cut-off value and genes were classified into those that, relative to mock-infected cells, were ≥2-fold up-regulated (+) or down-regulated (−) and those with no change in expression (fold change between +2 and −2).

In response to H1N1 infection, 100%, 100% and 95.84% of genes were concordantly expressed in the three donors at 1, 3 and 6 h post-infection respectively. Similarly, the concordance between donors of H5N1-infected cells at 1, 3 and 6 h post-infection was 99.87%, 99.74% and 92.34% respectively. Thus any variation in response to viral infection between donors was mainly seen at 6 h post-infection and for a minority of genes only. When the expression of those genes with discordant results between donors was further analyzed, in most instances they had the same trend of expression, being either up- or down-regulated in all donors and the differences reflected whether the cut-off of ≥2-fold change in gene expression compared to mock-infected cells was met. The genes that were found to be differentially regulated between donors were the gene G0/G1 switch 2 (G0S2) in H5N1-infected cells at 1 h post-infection and five genes (dual specificity phosphatase 1 (DUSP1), v-fos FBJ murine osteosarcoma viral oncogene homolog (FOS), glucosaminyl (N-acetyl) transferase 4 (GCNT4), microtubule associated serine/threonine kinase-like (MASTL), and TRAF family member-associated NF-κB activator (TANK)) in H5N1-infected cells at 6 h post-infection.

**Figure 1:**
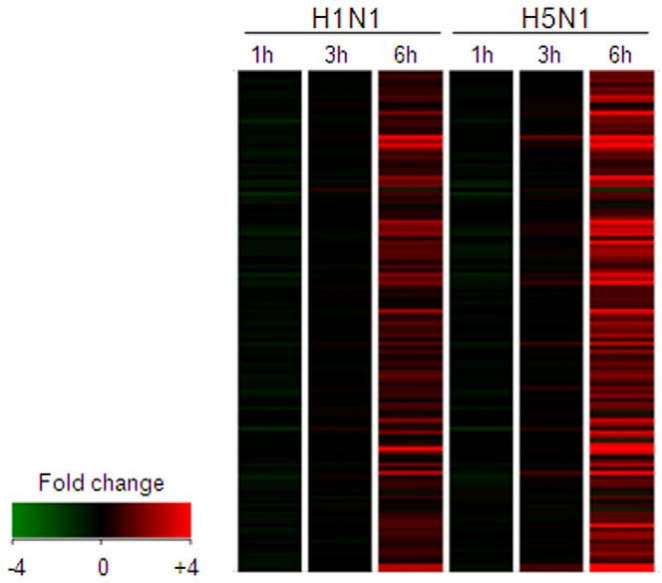
Heatmap showing microarray gene expression profiles of influenza A infected primary human macrophages at different post-infection time points. Expression of genes with p-values <0.05 and fold change ≥±1.5 in at least 1 of the 6 conditions in primary human macrophages infected with H5N1 or H1N1 viruses at 1, 3 and 6 h post-infection are shown. Note that the majority of gene expression changes were found at 6 h post-infection in both H5N1 and H1N1 infected cells. Data presented are averaged gene expression changes for three different individuals.

**Figure 2:**
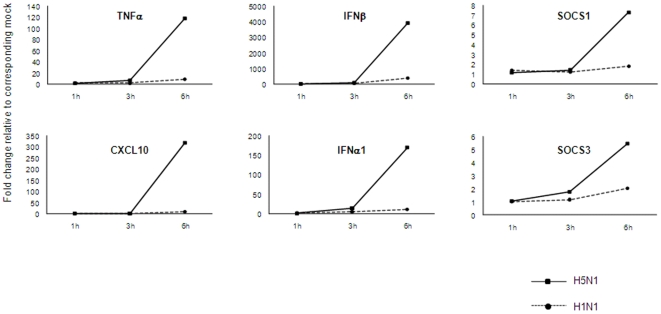
Validation of microarray data by real time PCR. Expression of six genes was assessed at 1, 3 and 6 h after infection by influenza A compared to mock infection. Data presented was from one representative donor and showed similar expression patterns compared with the microarray experiment.

Given the high overall concordance in gene expression profiles found among the three donors in our analysis, the fold change of gene expression levels in response to either H5N1 or H1N1 compared to mock infection was averaged from the three donors in the subsequent analysis. We filtered the averaged gene-expression data using a cut-off value of 1.5-fold up- or down-regulation in the H5N1- and H1N1-infected cells compared to mock. A total of 109 genes in H5N1-infected cells and 64 genes in H1N1-infected cells showed at least 1.5-fold difference in expression level compared to mock infected cells in at least one time point (Table S1). The majority of changes in gene expression occurred at 6 h post-infection in response to both viruses, with only five genes up-regulated at the 3 h time point in H5N1 infection and none at the 1 h time point (Table 1). Sixty genes were up-regulated in response to both H1N1 and H5N1 infection, and a heatmap revealed that the overall response to each virus appeared to be distinguished not by the genes that were differentially expressed, but rather by the intensity of expression (Figure 1). Of the 60 genes up-regulated in response to both viruses, 44 (73.3%) were up-regulated at least 1.5-fold more in response to H5N1 than H1N1. The expression levels of genes of particular interest, including TNF, chemokine (C-X-C motif) ligand 10 (CXCL10), IFN-α1 and -β1, and suppressor of cytokine signaling 1 and 3 (SOCS1, SOCS3), were verified by the use of real-time PCR, and the data are shown in Figure 2.

**Table 1:** Summary of genes differentially expressed in response to H1N1 and H5N1 infection.

	Time	Up	Down	Total
**H1N1**	**1 h**	0	0	0
	**3 h**	0	0	0
	**6 h**	64	0	64
**H5N1**	**1 h**	0	0	0
	**3 h**	5	0	0
	**6 h**	104	5	109

To compare the cellular response to H1N1 infection versus H5N1 infection, Gene Ontology and pathway over-representation analyses were performed on the gene sets described above using the tools provided in InnateDB (www.innatedb.ca), a freely-available database and analysis environment for the investigation of mammalian innate immune responses [17]. An independent analysis of the data was also carried out using GeneSpring software. As the latter analysis provided comparable results to InnateDB and did not contribute additional over-represented pathways, the Innate DB analysis is presented below.

The 64 H1N1-responsive genes and 109 H5N1-responsive genes were each submitted to InnateDB, and lists of Gene Ontology terms and pathways that were found to be significantly enriched (p-value <0.05 after multiple testing correction) in each gene set were generated. At the Gene Ontology term level, 19 GO terms were found to be enriched: 13 were common to both the H1N1 and H5N1 responses, two were unique to the H1N1 response, and four were unique to the H5N1 response (Table 2).

**Table 2:** Significantly enriched Gene Ontology terms in the response to H1N1 and H5N1 infection.

	H1N1		H5N1	
GO Term	Gene Count	p-value	Gene Count	p-value
apoptosis [BP]	7	8.45E-03	10	1.06E-03
blood circulation [BP]	---	NS	3	3.28E-02
cell motion [BP]	5	6.01E-04	5	3.81E-03
cell-cell signaling [BP]	14	6.62E-12	18	3.51E-14
chemokine activity [MF]	11	4.94E-15	11	1.08E-13
chemotaxis [BP]	12	1.80E-11	12	2.19E-09
cytokine activity [MF]	12	6.18E-12	15	8.80E-14
exocytosis [BP]	3	8.19E-03	3	2.50E-02
extracellular region [CC]	17	3.91E-03	22	3.77E-03
extracellular space [CC]	16	9.46E-10	22	9.50E-13
immune response [BP]	18	6.02E-12	23	8.71E-14
inflammatory response [BP]	16	1.55E-14	18	3.51E-14
interleukin-1 receptor binding [MF]	---	NS	2	4.38E-02
lipopolysaccharide-mediated signaling pathway [BP]	---	NS	2	2.66E-02
positive regulation of amino acid phosphorylation [BP]	2	3.03E-02	---	NS
protein import into nucleus, translocation [BP]	2	3.03E-02	---	NS
response to virus [BP]	7	4.63E-07	8	3.92E-07
signal transduction [BP]	15	4.93E-02	21	2.14E-02
tumor necrosis factor receptor binding [MF]	---	NS	4	1.83E-03

GO categories are denoted by BP (biological process), MF (molecular function), and CC (cellular component). Number of genes refers to those differentially expressed in the response to viral infection associated with a specific GO term. NS indicates a pathway was not enriched to a statistically significant degree in the condition in question.

At the pathway level, 17 pathways were enriched: 13 were common to both the H1N1 and H5N1 responses, three were unique to the H1N1 response, and one was unique to the H5N1 response (Table 3).

**Table 3:** Significantly enriched pathways in the response to H1N1 and H5N1 infection.

	H1N1		H5N1	
Pathway Name	Gene Count	p-value	Gene Count	p-value
Apoptosis	4/87	1.22E-02	5/87	2.01E-02
Canonical NF-κB pathway	2/22	4.11E-02	---	NS
Cd40l signaling pathway	2/9	1.15E-02	2/9	4.23E-02
Chemokine receptors bind chemokines	7/39	2.36E-08	7/39	1.76E-06
Cytokine-cytokine receptor interaction	13/266	8.34E-09	18/266	4.08E-10
dsRNA induced gene expression	2/14	2.25E-02	---	NS
Epithelial cell signaling in *H. pylori* infection	3/66	4.08E-02	---	NS
HIV-1 Nef: Negative effector of Fas & TNFα	---	NS	3/33	4.16E-02
IL1	3/32	1.06E-02	4/32	4.74E-03
IL12-mediated signaling events	4/59	6.88E-03	5/59	5.21E-03
IL23-mediated signaling events	3/36	1.17E-02	3/36	4.97E-02
JAK-STAT pathway and regulation pathway	4/95	1.42E-02	5/95	2.40E-02
NF-κB activation by *Hemophilus influenzae*	3/27	7.41E-03	3/27	2.73E-02
Signal transduction through il1r	3/35	1.21E-02	4/35	5.81E-03
TNFα	6/188	7.82E-03	7/188	2.14E-02
TNFR2 signaling pathway	2/12	1.77E-02	3/12	6.18E-03
Toll-like receptor signaling pathway	11/100	9.29E-11	12/100	2.23E-09

Number of Genes is provided as X/Y, where Y is the total number of genes in the pathway according to InnateDB, and X is the subset of those genes differentially expressed in the response to viral infection. NS indicates a pathway was not enriched to a statistically significant degree in the indicated viral treatment.

**Figure 3:**
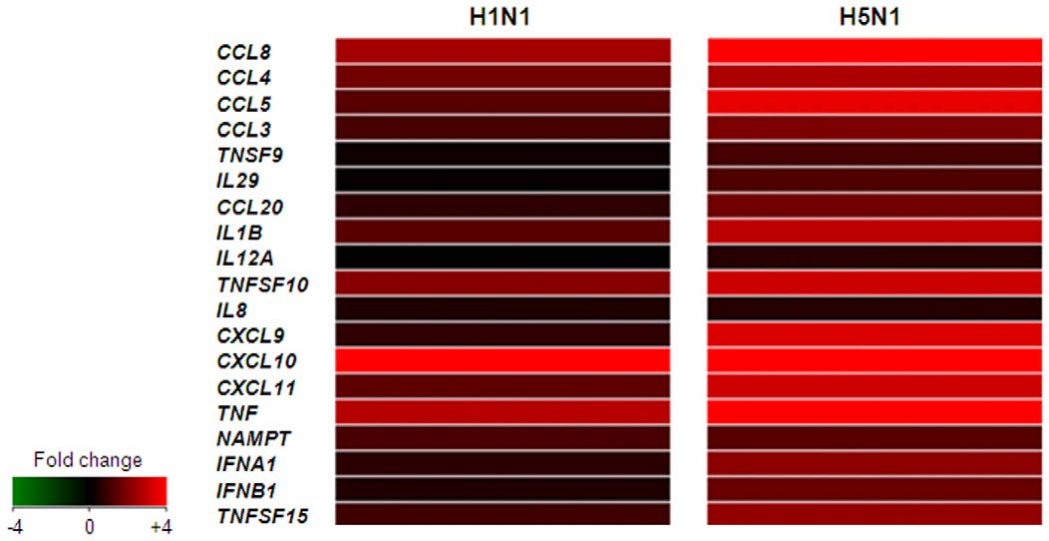
Expression of selected genes annotated are related to cytokine and chemokine activity. Increased gene expression levels were seen in response to H5N1 compared to H1N1 infection.

Over-representation analysis supported the earlier observation that the qualitative nature of the cellular response to H1N1 and H5N1 infection is similar, and that the differences lay instead at the quantitative level (Figure 1). Infection with both viruses resulted in the expression of genes associated with virtually identical functions, processes, and pathways and the few differences observed were likely to be artifacts of the statistical testing procedure. For example, the genes in the three pathways significantly enriched in response to H1N1 but not H5N1 were, in fact, also up-regulated >1.5-fold in response to H5N1, however the larger size of this geneset (109 versus 64 genes) reduced the statistical significance to a level >0.05. The response to both viruses is characterized by a strong chemokine/cytokine response, inflammation, a response to viral RNA, the involvement of TNF superfamily signaling pathways (IL1, IL12, IL23, TNF), and apoptosis, but the response elicited by H5N1 was greater than that induced by H1N1 virus. A heatmap of 19 genes with cytokine or chemokine activity further highlights the marked quantitative difference between the two conditions, with 17/19 chemokines/cytokines expressed at least 1.5-fold higher in the host response to H5N1 versus H1N1 (Figure 3).

### The Strong Type I IFN Host Response to H5N1 Infection

To further investigate differences in host response to H5N1 compared with H1N1 infection, we focused on the 63 host genes with fold changes that differed markedly (difference of ≥1.5fold) in response to the two viruses (Table 4). Four of these genes also showed >1.5 fold up-regulation in H5N1- compared with H1N1-infected cells at 3 h post-infection: TNF, IFN-induced protein with tetratricopeptide repeats 2 (IFIT2), macrophage inflammatory protein 1-β (CCL4L1), and phorbol-12-myristate-13-acetate-induced protein 1 (PMAIP1).

**Table 4:** Genes strongly up- or down-regulated in response to H5N1 infection versus H1N1 infection.

ATF5	CCNB1	GBP4	IFIT3	LAMP3	PPP1R15A	SOCS3
BAMBI	CCRN4L	GBP5	IFIT5	MX1	PTGS2	**TNF**
CCL20	CD274	GPR109A	IFNα1	MXD1	PTX3	TNFAIP3
CCL3	CMPK2	GPR109B	IFNβ1	NFKBIZ	RCAN1	TNFAIP6
CCL3L1	CXCL10	HERC5	IL1B	OASL	RNF19B	TNFSF10
CCL4	CXCL11	HIVEP2	IL29	OTUD1	RSAD2	TNFSF15
**CCL4L1**	CXCL9	IFIH1	INDO	PELI1	RTP4	TNFSF9
CCL5	DHX58	IFIT1	IRF1	PFKFB3	SGK1*	TRAF1
CCL8	DNAJB4	**IFIT2**	ISG15	**PMAIP1**	SOCS1	USP18

Fold changes of these 63 genes were at least 1.5-fold greater in H5N1-infected cells. All genes were up-regulated with the exception of SGK1, which was down-regulated. Genes in bold were also up-regulated to a greater degree at 3 h post-infection.

Gene Ontology and pathway over-representation analysis was then performed on this subset of genes, as well as the 49 genes differentially expressed in response to H5N1 only. Results from both genesets were similar to those obtained earlier with the full dataset, but now revealed the involvement of IFN signaling, including the JAK-STAT pathway.

Further inspection of the genesets revealed an unusually high number of IFN-responsive and/or related genes. Among the 63 genes, we identified two type I IFNs (IFN-α1, IFN-β1), the type III IFN interleukin 29 (IL29), several components of effector pathways governing the IFN-mediated antiviral response (including the IFN-induced 17kDa protein precursor ISG15, the Ubl carboxyl-terminal hydrolase-18 USP18, the 2′-5′oligoadenylate synthetase-like protein OASL, the myxovirus resistance protein 1 MX1, and the probable ATP-dependent RNA helicase DHX58), and a range of IFN-induced genes (including the IFN-induced with tetratricopeptide repeats proteins IFIT1, IFIT2, IFIT3, and IFIT5, and the IFN response factors IRF1 and IRF2). Interferome (http://www.interferome.org)[18], an IFN-regulated gene database, permitted the identification of 36 of the 63 (57.14%) H5N1 hyper-responsive genes as being related to the IFN response, while only 16 of the 50 genes (32%) with comparable responses to H5N1 and H1N1 were IFN-related (Table S1).

Recognizing that the stringent p-value cutoffs typically applied during microarray analysis can often obscure interesting trends in the data, we opted to perform a larger network-based analysis of the data to explore the potential involvement of the IFN pathway. We maintained a fold-change cutoff of 1.5, but expanded the dataset to include those genes with p-values between 0.05 and 0.1. This introduced 22 new genes into the analysis, 12 of which were IFN-related (Table 5.)

**Table 5:** Additional genes considered in the network-based analysis.

*C19orf66*	**DDX58**	**GBP7**	hsa-mir-155	*MYLIP*	***PTGER4***	**SPN**	**ZC3HAV1**
*C21orf91*	EREG	**GCH1**	***IL15RA***	**OAS2**	**RIPK1**	*SRGAP2P1*	
***CD40***	**GBP1**	GTF2B	*IL27*	**PPM1K**	SNORD45	TAGAP	

With the exception of SPN (-1.64 at 3 h post-H1N1), all genes were up-regulated. SNORD45 was only up-regulated in response to H1N1; the 8 italicized genes were only up-regulated in response to H5N1. Bolded genes are interferon-related and/or interferon-stimulated.

**Figure 4:**
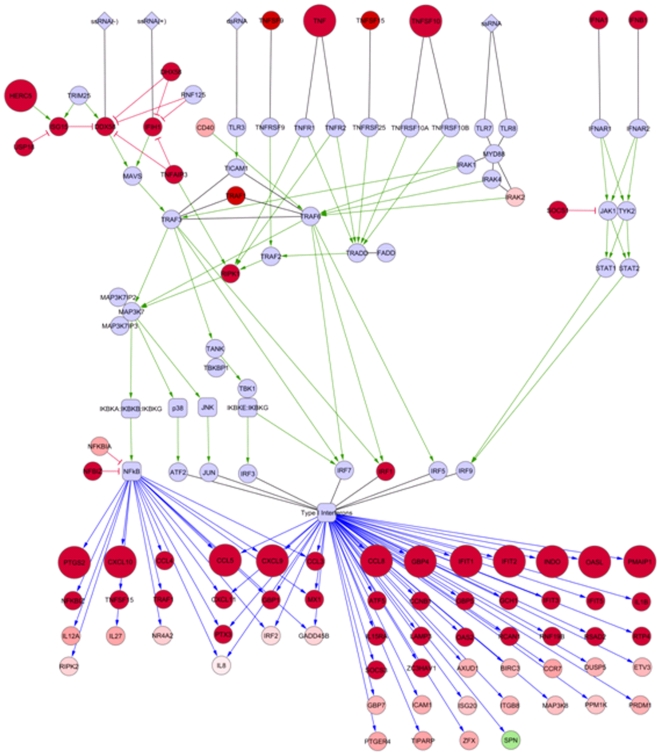
Components of the RIG-I, TNF and type I IFN pathways are up-regulated in response to H5N1 to greater extent than H1N1. Viral ligands are shown as diamonds, genes/proteins as circles, and complexes or pathways as rectangles. Green arrows indicate activation, red T-bars represent inhibition, black lines indicate binding, and blue lines indicate stimulation of gene expression. For clarity, blue lines feeding back to upstream pathway components were removed; many of these upstream components, however, are regulated by type I IFN and/or NFκB. The colour of a node reflected the H5N1:H1N1 expression ratio: blue nodes were not significantly differentially expressed in response to either virus, pink/red nodes were up-regulated more in response to H5N1 than H1N1 (red  = >1.5-fold more in response to H5N1, pink  = 1.0-1.49-fold higher in response to H5N1), and the green node was down-regulated to a greater extent in response to H5N1. Larger nodes had a fold-change value of >10 in H5N1 infection.

Using pathway and interaction information from the literature and the InnateDB database, we constructed, using Cytoscape [19], a network illustrating the cross-talk between the IFN, TNF and RIG-I pathways, all of which were implicated by our analyses in the response to H5N1 infection – (Figure 4); the colour and size of nodes reflect the degree of fold-change difference between the H5N1 and H1N1 response. Pathway ligands, such as TNF and IFN family members, represent the bulk of up-regulated genes in the pathways, while the signaling components generally remain unchanged – consistent with the fact that the activity of many of these is controlled not through transcriptional regulation but rather by phosphorylation status. Several inhibitory feedback mechanisms of the RIG-I antiviral pathway, however, were strongly up-regulated. There was, for example, a strong up-regulation of SOCS expression in response to H5N1 infection, which may lead to the inhibition of the anti-viral IFN signaling.

## Discussion

To elucidate the mechanisms of pathogenesis of highly pathogenic avian influenza H5N1 in humans, we identified here the commonalities and differences of host-response signaling pathways in primary human macrophages infected with highly pathogenic influenza A/Vietnam/3212/2004 (H5N1) compared to a low-pathogenicity seasonal human influenza A/Hong Kong/54/1998 (H1N1). These two viruses were selected as they are representative of HPAI H5N1 and seasonal human influenza viruses in general with respect to cytokine phenotype [20]. We carried out a comprehensive microarray study to compare the host responses of H5N1- and H1N1-infected primary human macrophages at different stages of the viral replication cycle (1, 3 and 6 h post-infection).

Cells isolated from three individual donors were used as biological replicates. In general, we observed a high concordance of gene expression profiles among different donors' cells in response to influenza infection. The exceptions found were the genes G0S2, DUSP1 and FOS, which are believed to have important roles in cell cycle and cellular proliferation/differentiation in various cell types [21], [22], [23], and TANK, which has an essential role in type I IFN production [24]. Whether these genes are more affected by genetic polymorphisms in humans remains to be determined.

When comparing gene expression profiles of H5N1- or H1N1-infected cells versus mock-infected cells, most of the genes (60/64) found to be significantly up-regulated (fold-change >1.5) after H1N1 infection were also up-regulated in response to H5N1 infection, although nearly three-quarters of these genes were expressed >1.5-fold more in response to the avian influenza virus. Gene Ontology and pathway over-representation analysis also indicated that both viruses elicited similar host responses in terms of signaling pathways, molecular functions and biological processes, with an enrichment of cytokines/chemokines, immune and inflammation-related pathways, including Toll-like receptor signaling, TNF signaling, JAK-STAT pathway, and apoptosis. This similarity leads us to conclude that differences in host responses to these viruses are primarily quantitative rather than qualitative in nature. This quantitative difference is unlikely to be due to differences in virus replication competence, because we were able to show that the levels of viral M gene for both H1N1 and H5N1 infection are comparable, indicting that there are no major differences in viral gene transcription between these viruses.

xTo further investigate the quantitative difference, we next examined a set of 63 genes that were differentially expressed in response to H5N1 to a level at least 1.5-fold greater than their expression in response to H1N1. Gene Ontology and pathway over-representation analysis yielded many of the same pathways and processes as did the earlier analysis of the complete geneset; however the involvement of the type I IFN pathway in the H5N1 response became more evident. Recognizing that the ontology and pathway annotations stored in InnateDB often capture only canonical ontology pathway members and may not include all related genes, we used further information from the literature and the Interferome database of IFN-regulated genes to construct a more comprehensive network showing the cross-talk between the type I IFN pathways and the pathways that lie upstream of the type I IFN genes that were identified in our analysis, including TLR signaling, other viral sensing pathways, and TNF signaling.

### Type I IFN Responses and Signaling through JAK-STAT Is Enhanced in Response to H5N1 Infection

In this study, we found that 52 out of the total of 113 differentially expressed genes were either IFNs or IFN-stimulated genes (64/134 when genes with p-values between 0.05 and 0.1 are considered), and that 36 of these were expressed to a higher degree (>1.5-fold vs H1N1) in response to H5N1 infection.

For instance, the gene expression data indicates an increased expression of both IFN-α1 and IFN-β at 6 h after H5N1 infection, although only modest increases of both IFNs are seen in response to H1N1. We also observe the up-regulation of IL29 at 6 h following H5N1 infection, but not H1N1 infection. IL29 was recently recognized as a type III IFN, which signals through a similar JAK-STAT pathway as type I IFNs [25].

Type I and III IFNs bind to the IFNAR1/IFNAR2 and IL10R2/IFNλR1 receptors respectively. Upon binding of IFNs, the corresponding receptor subunits dimerize to form the receptor complex and activate JAK-STAT signaling, which then results in downstream induction of a range of genes through ISGF3, a trimeric transcription factor complex of signal transducer and activator of transcription 1 and 2 (STAT1 and STAT2) and IRF9. Downstream genes regulated by ISGF3 include viral sensors, antiviral effector molecules, inhibitors of IFN signaling and viral sensing, and a range of other IFN-stimulated genes, or ISGs.

Amongst the IFN-stimulated genes, we found both positive and negative effectors. A number of genes implicated in the antiviral response are up-regulated, many to a much higher degree in H5N1 infection, and include the 2′-5′-oligoadenylate synthetases OASL and OAS2, several guanylate binding proteins (GBPs 1, 4, 5 and 7), myxovirus resistance protein 1 (MX1), radical S-adenosyl methionine domain containing protein 2 (RSAD2), pentraxin related protein PTX3, IFN-induced protein with tetratricopeptide repeats 1 (IFIT1), and zinc finger CCCH-type antiviral protein 1 (ZC3HAV1). Similarly, a number of IFN-stimulated pro-inflammatory cytokines are also up-regulated, including IL1B, CXCL9 and 11, and CCL5 and 8.

SOCS1 and SOCS3 induction, for example, was particularly robust, with nearly 4-fold up-regulation 6 h after H5N1 infection but only a 1.4-fold change 6 h following H1N1 infection. SOCS proteins are known to down-regulate Toll-like receptor signaling and IFN signaling [26], [27], [28], [29], [30] and were recently implicated in the modulation of H3N2-induced innate immune responses in human lung epithelial cells, where over-expression of SOCS1 in H3N2-infected cells significantly reduced activation of the antiviral IFN-β pathway, but enhanced activation of the pro-inflammatory NFκB pathway [31]. SOCS3 over-expression inhibited both IFN-β and NFκB. Our finding that only SOCS1 and SOCS3 were over-expressed is in agreement with Pothlichet et al., who observed an identical pattern of up-regulation in response to H3N2, which suggested that both SOCS proteins act in concert to inhibit IFN signaling and therefore inhibit the host's anti-viral responses in the later stage after H5N1 infection.

IRF2 is one of only two IFN transcriptional regulatory factors up-regulated in the present study – IRF1 is the other – and is up-regulated in response to H5N1 infection at 6 h. IRF2 is a transcriptional repressor that inhibits IRF1 [32] and has not previously been implicated in influenza virus infection. While the up-regulation of this repressor may again be part of the cell's attempt to limit a hyperactive IFN response, IRF2 may also be another pro-inflammatory mechanism unique to H5N1, as IRF2 was recently demonstrated to enhance NFκB activity via recruitment of the RELA (p65) subunit to the nucleus [33].

On the other hand, upstream of IFN signaling, we also observed what appears to be an attempt by the host to shut down the pathways governing the production of type I IFN at 6 h post-infection time. Specifically, we note the up-regulation of the NFκB inhibitors NFKBIA and NFKBIZ, and the up-regulation of several negative regulators of the innate sensing receptor retinoic acid-inducible gene 1 (RIG-I)-like receptors (RLRs), including USP18, ISG15, DHX58 and tumor necrosis factor alpha-induced induced protein 3 (TNFAIP3). Our data here suggested that at the early stage after influenza infection, the cells response by up-regulation of the IFN and IFN signaling as part of the antiviral mechanism. Activation of various IFN regulatory mechanisms such as up-regulation of negative regulators, SOCS via JAK-STAT signaling may lead to the suppression of IFN and/or other anti-viral gene expression in the later stage of virus infection and therefore provide the virus with additional time to replicate. The increase in viral load can cause infected cells to produce excessive pro-inflammatory cytokines with the additional infiltration of immune cells and finally lead to severs tissue damage. This may also be true to explain the pathogenicity of 1918 virus which its NS1 gene has suggested to block the IFN-stimulated gene expression during later stage of infection [34].

### Parallel Activation of TNF-α Signaling Results in a Synergistic TNF/IFN Response in H5N1-Infected Cells

In parallel with the type I IFN-mediated activation of the JAK-STAT pathway, H5N1 also causes TNF-mediated activation of a range of downstream effectors. The two major signaling cascades induced by TNF are apoptotic and inflammatory signaling pathways. Many downstream targets of TNF are also stimulated by type I IFN, as was demonstrated in a recent microarray study comparing cellular responses to treatment with each cytokine [35].

We previously demonstrated that TNF-α was hyper-induced in autopsy lung tissue from patients with H5N1 disease [9] and in H5N1-infected human macrophages in vitro as early as 3 h post-infection [14] and have suggested that increased production of pro-inflammatory cytokines in response to H5N1 compared to H1N1 infection may be an important factor contributing to H5N1 pathogenesis. Here, we have demonstrated that TNF-α expression was significantly up-regulated as early as 3 h post-infection in response to H5N1 compared to H1N1 infection. At 6 h post-infection this trend continued, and several other members of the TNF-α superfamily were also up-regulated: TNFSF9, TNFSF10 and TNFSF15 (all at least 1.7-fold higher in H5N1-treated cells). This enhanced expression of ligands is likely the primary force driving pathway activation, and is manifested in dramatically increased expression levels of many pro-inflammatory chemokines. CXCL9, CCL5, and CXCL10, for example, were differentially upregulated by H5N1 6.2-, 4.4-, and 3.8-fold higher than H1N1, respectively, and all three exhibited expression levels over 10-fold higher than mock-infected cells.

We recently reported that TNF-α, IFN-β and IL-29 are primary cytokines potently induced by the H5N1 virus, which then act by autocrine and paracrine pathways to activate other mediators in the cytokine cascade [15]. This contention is supported by the current study. Of the five genes differentially expressed in response to H5N1 at the 3 h time point, TNF-α appears to be a likely candidate for inducing a hyper-inflammatory response in H5N1 infection. It was recently demonstrated that TNF induces a sustained and elevated inflammatory response in macrophages: TNF-α stimulation results in the expression of pro-inflammatory genes and higher expression of the IRF1 transcription factor [36]. IRF1 activates JAK-STAT signaling to produce IFN-β, pro-inflammatory cytokines, and signaling adaptors that result in an autocrine loop. Although this study took place over a longer time period than the present work, it is notable that TNF ligands and IRF1 were both up-regulated in response to H5N1, and that many of the pathways implicated by Yarilina et al were active in our dataset.

A later microarray analysis of genes stimulated in response to TNF, IFN-β, or both revealed a synergistic state whose extent had not previously been recognized [35]. The authors of this latter study demonstrated that there was a distinct set of genes only up-regulated in the presence of both cytokines (3 of these 8 genes are up-regulated in the present study: CCL5, DHX58 and GBP4), as well as a set of shared genes that are expressed to a modest degree in response to both of the cytokines administered separately, but a markedly up-regulated degree in response to the two cytokines administered together (11 of these 25 genes were up-regulated in our dataset).

Observations by others that TNF and type I IFN, specifically IFN-β, synergistically work together to alter the kinetics of gene expression provides a potential model for H5N1's enhanced pathogenesis. In macrophages, the virus causes early induction of TNF-α and IFN-β, whereas H1N1 infection also results in an induction of both TNF-α and IFN-β but with a much lower magnitude. The synergy between TNF-α and IFN-β results in a more pronounced IFN and pro-inflammatory cytokine response after H5N1 infection, and the massive up-regulation of these cytokines tips the balance in favour of a hyper-inflammatory response.

### Apoptosis

TNF-α signaling can exert both pro-apoptotic effects and anti-apoptotic NFκB-dependent mechanisms that trigger cell survival, with the anti-apoptotic activities of TNF-α regarded to be its dominant effect. The apoptotic signaling pathway is mediated by TNF receptor I (TNFR1) via the intermediate adapter TNF receptor-associated death domain protein (TRADD), which activates caspase 8 and triggers the downstream caspase cascade via mitochondria-dependent and independent mechanisms. The inflammatory arm of TNFR1 signaling is also mediated via TRADD, which recruits TNF receptor-associated factor 2 (TRAF2). The association of TRAF2 and RIPK1 activates MAPK pathways, which in turn lead to the activation of NFκB, a key transcription factor inducing many target genes, including many components of the inflammatory and cell survival responses [37].

From the microarray data in the present study, we have identified that PMAIP1, (also named APR or NOXA) was differentially induced in response to H5N1 compared with H1N1 as early as at 3 h post-infection. PMAIP1 is a Bcl-2 homolog 3 (BH3) containing member of the Bcl-2 family of proteins [38]. PMAIP1 has been reported to be induced by p53 [38], UV radiation [39], IFN, dsRNA and viral stimulation [40] and is proposed to mediate apoptosis by interacting with other pro- or anti-apoptotic Bcl-2 family members (such as Bax and Bak) in a direct and indirect manner to promote mitochondrial membrane changes, leading to membrane permeabilization and efflux of apoptogenic proteins, thus promoting apoptosis [41], [42]. However the exact mechanisms of PMAIP1-regulated apoptosis are still not yet well-defined.

The role of apoptosis in viral replication may vary with different viruses. For example, in certain acute viral infections, lytic viruses can utilize host apoptotic cascades to aid in cell lysis and virus dissemination. In such cases, the infected cell releases the infectious viral particles as well as the immature unpacked viral material including dsRNA to the micro-environment. The dsRNA can trigger innate sensing receptors of neighboring cells and trigger apoptosis in these cells which otherwise may have hosted additional cycles of virus replication. This is an example of the host initiating “altruistic” apoptosis in bystander cells which can lead to limiting viral spread [43]. Taken together, further investigation of the pathogenic significance of the early expression of PMAIP1 in response to H5N1 infection observed in our microarray data is necessary. The role of PMAIP1 in apoptosis, as well as whether apoptosis or delayed apoptosis contributes to H5N1 pathogenesis through its positive or negative effects on viral replication, remain to be determined. Nevertheless, we have previously shown that compared with H1N1 virus, H5N1 virus-infected macrophages were found to have delayed apoptosis [44].

### Comparison with Microarray Data from Experimental Animal Infection

Recently, similar global gene expression profiling studies utilizing H5N1-infected ferret and primate models were reported [10], [45]. In the ferret model, IFN response genes as well as the pro-inflammatory cytokine and chemokine genes were found to be hyper-induced in H5N1-infected ferret lungs compared to the lungs from the less pathogenic H3N2-infected animals, and the authors hypothesized that despite the expression of inhibitory genes, this hyper-induction and persistent IFN and pro-inflammatory response might form the basis of H5N1 pathogenesis. This hypothesis was supported by the observation that blocking of the CXCL10 signaling pathways via CXCR3, CXCL10's cognate receptor, led to a reduction of symptom severity as well as delayed mortality. Similarly, H5N1-infected primates exhibited severe bronchiolar and alveolar lesions and the expression and production of type I IFN and pro-inflammatory cytokines such as TNF-α, IL6 and CXCL10 were found to be markedly higher in H5N1-infected lungs.

Data from animal studies cannot differentiate whether the observed effects were due to intrinsic differences in host response induced by the H5N1 virus (versus seasonal influenza) or whether they reflect the greater viral replication competence or even whether they merely secondarily reflect enhanced tissue damage. Our data, which arises from a single synchronous infection of cells with an equivalent virus dose, proves that these host responses are driven by intrinsic differences of the H5N1 virus.

### Conclusion

Our gene expression analysis of primary human macrophages infected with comparable virus doses of the high-pathogenicity H5N1 and low-pathogenicity H1N1 viruses reveals differences at the quantitative rather that at the qualitative levels. An up-regulation of pathway ligands rather than signaling intermediaries appears to be the driving force behind pathway activation in the response to H5N1. However, a role for differential phosphorylation of signaling intermediaries and other regulatory mechanisms, such as post-transcriptional regulation by microRNAs, cannot be excluded as a contributory mechanism for activation of host responses. Integration of data from complementary approaches will be required in the future to more fully understand the molecular events that lead to viral pathogenesis.

The H5N1-induced hyper-activation of IFN-β and TNF-α pathways synergize to generate a highly pro-inflammatory cytokine/chemokine response that is markedly elevated compared to H1N1 infection and likely tips the delicate balance of the innate immune system towards inflammation, thereby contributing to tissue damage. We also observed an increase in inhibitors of certain pathways (e.g. SOCS), which may reflect the cell's attempt to shut off these pathways that have been over-activated in response to H5N1, but which may themselves result in a skew of the innate immune response to one of an increased pro-inflammatory nature.

It may eventually be possible to exploit these quantitative differences in host pathways activated by H5N1 for therapeutic purposes through the use of the appropriate inhibitors. Further investigation into the nature of these inhibitors and their specific targeting mechanisms is a challenging but critical task, and may ultimately result in novel therapeutic strategies that are capable of restoring the equilibrium of the host response and minimizing the impact of H5N1 infection on the host.

## Materials and Methods

### Ethics Statement

The research protocol of using primary human macrophages in this study was approved by the research ethics committee of the University of Hong Kong.

### Viruses

The viruses used were A/Vietnam/3212/2004 (H5N1), an influenza virus isolated from a patient with fatal H5N1 disease in Vietnam during 2004, and the human seasonal influenza virus A/Hong Kong/54/1998 (H1N1). From their initial isolation, these viruses were propagated in Madin-Darby canine kidney (MDCK) cells. Virus infectivity was determined by cytopathic assays on MDCK cells and quantified as tissue culture infectious dose 50 (TCID50) [14].

### Primary Human Macrophage Culture

Peripheral-blood leucocytes were separated from buffy coats of healthy blood donors (provided by the Hong Kong Red Cross Blood Transfusion Service) by centrifugation on a Ficoll-Paque density gradient (Pharmacia Biotech) and purified by adherence [14]. Macrophages were seeded onto tissue culture plates in RPMI 1640 medium (Sigma-Aldrich) supplemented with 5% heat-inactivated autologous plasma. The cells were allowed to differentiate for 14 days in vitro before use. No exogenous cytokines or growth factors were added to aid cell differentiation.

### Virus Infection of Macrophages

Differentiated macrophages were infected with H1N1 and H5N1 at a multiplicity of infection (MOI) of two. After 30 min to allow virus adsorption, the inoculum was removed, the cells were washed with warm culture medium and incubated in macrophages serum free medium (SFM) (Invitrogen) supplemented with 0.6 mg/l penicillin and 60 mg/l streptomycin. Mock infected cells served as controls. Total RNA was extracted from cells after 1, 3, and 6 h post-infection using the RNeasy Mini kit (Qiagen) according to the manufacturer's recommended protocol.

### Microarray Analysis

Human gene expression was examined with the GeneChip Human Gene 1.0 ST array (Affymetrix). The Human Gene 1.0 ST array comprises more than 750,000 unique 25-mer oligonucleotide features, constituting over 28,000 gene-level probe sets. RNA quality control, sample labeling, GeneChip hybridization and data acquisition were performed at the Genome Research Centre, The University of Hong Kong. The quality of total RNA was checked by the Agilent 2100 bioanalyzer. The RNA was then amplified and labeled with GeneChip® WT Sense Target Labeling and Control Reagents kit (Affymetrix). cDNA was synthesized, labeled and hybridized to the GeneChip array according to the manufacturer's protocol. The GeneChips were finally washed and stained using the GeneChip Fluidics Station 450 (Affymetrix) and then scanned with the GeneChip Scanner 3000 7G (Affymetrix).

GeneSpring GX 10.0 (Agilent) was used for the normalization, filtering and statistical data analysis of the Affymetrix microarray data. Briefly, normalization was performed using Exon Robust Multichip Average (RMA) algorithm. Probesets with an intensity value of the lowest 20th percentile among all the intensity values were removed. The remaining entities, with intensity values between the 20th and 100th percentile, resulted in a working transcript list used for statistical analysis. An analysis of variance (ANOVA), using Benjamini-Hochberg multiple testing correction, was performed to identify genes significantly differentially expressed (p<0.05) in response to virus infection in at least one time point. Significantly differential expressed genes with fold change ≥1.5 were then merged into a gene list for further Gene Ontology (GO) and pathway analysis.

GO and pathway over-representation analysis as well as further analysis of protein:protein and other interactions and were carried out using the InnateDB platform (http://www.innatedb.ca) [17]. Over-representation analyses were performed using default parameters (hypergeometric algorithm, Benjamini-Hochberg multiple testing correction). Results with p-values of <0.05 after multiple testing correction were considered to be statistically significant, and results with only a single GO term or pathway were not considered. IFN-response related genes were extracted from the Interferome database [18]. Molecular interaction networks were visualized using Cytoscape [19]. In parallel, an independent pathway over-representation analysis was also performed using the GeneSpring program. Human pathway databases, including Integrating Network Objects with Hierachies (INOH), Reactome, Kyoto Encyclopedia Genes and Genomes (KEGG), Biocarta, National Cancer Institute (NCI) and NetPath, were imported into the software for pathway over-representation of statistically significant genes.

### Real-Time Quantitative RT-PCR Assays

Total RNA was isolated using the RNeasy Mini kit (Qiagen) as described above. The cDNA was synthesized from mRNA with poly(dT) primers and Superscript III reverse transcriptase (Invitrogen). Transcript expression was monitored using a Power SYBR® Green PCR master mix kit (Applied Biosystems) with corrsponding primers. The fluorescence signals were measured using the 7500 real-time PCR system (Applied Biosystems). The specificity of the SYBR® Green PCR signal was confirmed by melting curve analysis. The method used for quantifying mRNA has been decribed elsewhere [15].

### Microarray Data Accession Number

All data is MIAME compliant and has been deposited in the Gene Expression Omnibus (GEO) database (www.ncbi.nlm.nih.gov/geo/) with the accession number: GSE 18816.

## Supporting Information

Table S1 Summary of gene expression in response to influenza A virus infection. Fold change of gene expression in response to H1N1 and H5N1 compared to mock infection at 1, 3 and 6 h post-infection time were shown. The “-” and no sign before the number indicates the down- and up-regulation of the gene respectively in influenza A infected cells compared to mock. HGNC Gene Symbol is HUGO Gene Nomenclature Committee approved gene symbol. *Ratio [H5N1,6 h]/[H1N1,6 h] indicates the fold change of gene expression in response to H5N1 compared to H1N1 infection at 6 h post-infection time. **IFN-Related? indicates if the gene is related to the IFN response.
(0.05 MB XLS)
